# Draft Genome Sequence of Aneurinibacillus migulanus Strain Nagano

**DOI:** 10.1128/genomeA.00232-15

**Published:** 2015-04-02

**Authors:** Faizah N. Alenezi, Hedda J. Weitz, Lassaad Belbahri, Hassen Ben Rebah, Lenka Luptakova, Marcel Jaspars, Stephen Woodward

**Affiliations:** a Biological Interaction in Soils, Institute of Biological and Environmental Sciences, University of Aberdeen, Aberdeen, United Kingdom; b Laboratory of Soil Biology, Department of Biology, University of Neuchâtel, Neuchâtel, Switzerland; c NextBiotech, Agareb, Tunisia; d Marine Biodiscovery Centre, Department of Chemistry, University of Aberdeen, Aberdeen, United Kingdom

## Abstract

Aneurinibacillus migulanus is characterized by inhibition of growth of a range of plant-pathogenic bacteria and fungi. Here, we report the high-quality draft genome sequences of A. migulanus Nagano.

## GENOME ANNOUNCEMENT

Plant diseases are responsible for many economic losses in landscape, agriculture, and forest settings through effects on visual amenity and decreasing yields and quality of crops. Infected food may also contain mycotoxins that result in poisoning or death of humans and other animals. Plant pathogens can cause huge losses in the production of individual crops, in certain instances between 25 and 100% (1, 2). Producing food that is free from toxic chemicals and maintaining a healthy environment are the main reasons to promote the development of environmentally sound methods of disease control, such as the use of biological control agents that can suppress pathogen activities (3). One bacterium with potential as a biological control agent is Aneurinibacillus migulanus, a Gram-positive, rod shaped, and spore-forming bacterium producing the antifungal/antibacterial metabolite gramicidin S, which acts directly against spore germination and the growth of pathogens, such as Botrytis cinerea (4, 5). It has also been suggested that A. migulanus Nagano produces biosurfactants that increase the rate of evaporation from plant surfaces, reducing periods of surface wetness and thereby indirectly inhibiting spore germination (4, 6).

The genome of A. migulanus Nagano was sequenced using the bacterial genome annotation system BG7, which was specifically designed for next-generation sequencing (NGS) data (Era7 Bioinformatics, Granada, Spain [7]). We obtained approximately 14.62 million reads for assembly after the low-quality reads were filtered out. The whole genome was *de novo* assembled into 82 contigs (*N*_50_, 195,382 bp) and rearranged into 175 scaffolds.

The draft genome sequence of A. migulanus Nagano consists of 5,959,194 bases, with the largest contig of 638,342 bp and 43.04% G+C content. The Nagano genome contained sequences for the synthesis of 4,817 proteins, of which 1,114 proteins were uncharacterized.

### Nucleotide sequence accession number.

The draft genome sequence of A. migulanus Nagano was deposited in GenBank under the accession no. JYBN00000000. This paper describes the first version of the genome.
